# Lipoprotein Lipase SNPs rs13702 and rs301 Correlate with Clinical Outcome in Chronic Lymphocytic Leukemia Patients

**DOI:** 10.1371/journal.pone.0121526

**Published:** 2015-03-26

**Authors:** Ans Rombout, Basile Stamatopoulos, Laurence Lagneaux, Sofie Lust, Fritz Offner, Evelien Naessens, Hanne Vanderstraeten, Bruno Verhasselt, Jan Philippé

**Affiliations:** 1 Department of Clinical Chemistry, Microbiology and Immunology, Ghent University Hospital, Ghent University, Ghent, Belgium; 2 Laboratory of Clinical Therapy, Institut Jules Bordet, Université Libre de Bruxelles (ULB), Brussels, Belgium; 3 Department of Hematology, Ghent University Hospital, Ghent University, Ghent, Belgium; Queen's University Belfast, UNITED KINGDOM

## Abstract

Chronic lymphocytic leukemia (CLL) is the most common leukemia in the Western world and is characterized by a heterogeneous clinical course. This variability in clinical course has spiked the search for prognostic markers able to predict patient evolution at the moment of diagnosis. Markers demonstrated to be of value are the mutation status of the immunoglobulin heavy chain variable region genes (*IGHV*) and lipoprotein lipase (*LPL*) expression. High *LPL* mRNA expression has been associated with short treatment free (TFS) and decreased overall survival (OS) in CLL. The *LPL* SNPs rs301 (T<C), rs328 (C<G) and rs13702 (T<C) have been associated with various metabolic disorders, but the association with CLL evolution is unknown. Here, in a cohort of 248 patients, we show that patients with the *LPL* SNP rs13702 wild-type T/T genotype had significantly shorter OS than patients with C/C and T/C genotypes (median time until CLL related death: 90 and 156 months respectively, *p*=0.008). The same was observed for *LPL* SNP rs301 (median time until CLL related death T/T: 102 and C/C, T/C: 144 months, *p*=0.03). Both SNPs rs301 and rs13702 were significantly associated with each other and notably, no association was found between *IGHV* status and presence of the SNP genotypes, indicating that these *LPL* SNPs are reliable prognostic markers that could add extra prognostic and predictive information to classical markers and help to improve the management of CLL.

## Introduction

Chronic lymphocytic leukemia (CLL) is the most common leukemia in the Western world and mainly affects elderly people [1]. Given its highly variable disease course, prognostic markers that allow for estimating the risk of disease progression and prognosis have been identified. Interestingly, lipoprotein lipase (*LPL*) was among the most differentially expressed genes reported in the initial profiling studies of Rosenwald et al. and Klein et al. [2,3]. We and others have shown that *LPL* mRNA expression is associated with short treatment free (TFS) and decreased overall survival (OS) in CLL [4–6]. How LPL contributes to a worse outcome in CLL and which mechanisms regulate its expression in CLL cells, remains to be elucidated.

Although the genetic basis of CLL is largely unknown, strong evidence suggests that a genetic component contributes to the etiology of this disease. Approximately 10% of patients have a family history of CLL, suggesting an inherited predisposition [7,8]. Genome-wide association studies (GWAS) have shown that “common variants”, single nucleotide polymorphisms (SNPs) with a population prevalence of at least 1%, contribute to this heritable risk of CLL [9–11]. The *LPL* SNPs rs301 (T<C), rs328 (C<G) and rs13702 (T<C) have been associated with various metabolic disorders, such as insulin resistance and atherosclerosis [12,13], but the association with CLL disease evolution is unknown. Our aim was to evaluate whether these variants in the *LPL* gene are associated with the clinical course of CLL patients.

## Materials and Methods

### Ethics Statement

This study was approved by the Ghent University Hospital Ethics Committee and conducted according to the principles expressed in the Declaration of Helsinki. Patient samples were obtained after written informed consent.

### Patients and Sample Collection

248 patients diagnosed with CLL were included in this study. The diagnosis and clinical stage for all patients were confirmed. Clinical stage was determined using the Binet staging system. Flow cytometric analysis of CD38 and zeta-chain associated protein kinase of 70 kDa (ZAP70), *IGHV* sequencing and cytogenetic characteristics were determined for the majority of patients as previously described [14,15]. OS was defined from the date of diagnosis to the death of the patient or the date of last follow-up before death. TFS was defined as the period between diagnosis and first CLL-specific treatment. Treatment was started when the lymphocyte count exceeded 10^11^ cells/L, or when patients developed massive lymphadenopathy, anemia, thrombocytopenia, splenomegaly or infections attributed to CLL related immune defects. Inclusion of patients was based on the availability of biological samples. Peripheral blood mononuclear cells (PBMCs) in fetal bovine serum (Hyclone, Thermofisher Scientific, Waltham, MA, USA) with 10% DMSO (Sigma-Aldrich, Diegem, Belgium) stored in liquid nitrogen were available from 169 patients. For the remaining 79 patients white blood cells stored at -80°C in ethanol were available.

### Selection of *LPL* SNPs

Three SNPs, rs301, rs328 and rs13702 located in different regions of the *LPL* gene were selected in this study, based on the minor allele frequency (MAF) in Caucasians, availability of Taqman SNP genotyping assays and reports in literature. The location of the SNPs in the *LPL* gene and their MAFs in Caucasians are shown in Table 1.

**Table 1:** Overview of examined genetic variants in the *LPL* gene.

			Caucasian	
dbSNP rs number		SNP location	MAF exp	MAF obs
rs301	T<C	Intron 6	0.25	0.24
rs328	C<G	Exon 9	0.13	0.12
rs13702	T<C	3'UTR	0.29	0.30

Both expected (exp) and observed (obs) minor allele frequencies (MAF) are shown for Caucasians. All variants are in accordance with Hardy Weinberg law. rs indicates referenced SNP id number; T<C indicates a transition of T to C nucleotide; C<G indicates a transversion of C to G nucleotide; UTR indicates untranslated region.

### Genotyping

gDNA was extracted using the *wizard SV 96 genomic DNA* purification system (Promega, Leiden, The Netherlands). In case of a low yield, pre-amplification of gDNA was performed with the GenomiPhi V2 *DNA Amplification* Kit (GE Healthcare, Diegem, Belgium). 10 ng of gDNA was used in Taqman SNP genotyping assays (Applied Biosystems, Life Technologies, Merelbeke, Belgium) for SNPs rs301, rs328 and rs13702 on an ABI Prism 7300 Real Time PCR System (Applied Biosystems, Life Technologies).

### RNA Isolation, cDNA Synthesis and Real-Time Quantitative PCR

Total cellular RNA was extracted with the miRNeasy mini kit (Qiagen, Hilden, Germany) according to the supplier’s instructions. Contaminating DNA was removed through DNase treatment using the DNase I, Amplification Grade, kit (Life Technologies). Determination of *LPL* expression levels was performed as previously described by Van Bockstaele et al. [4]. PCR reactions were performed with the LightCycler 480 Probes Master mix (Roche Diagnostics, Vilvoorde, Belgium) as a reaction mix, in a final volume of 15 μl in 384-well plate (Roche Diagnostics). All reactions were done in duplicate, and each PCR run included controls and a calibration curve of 6, 2-fold dilutions of cDNA from the HL-60 cell line (ATCC). Two housekeeping genes, *ACTB* (primers described by [16]) and *ABL1* (primers and probe described by [17]) were used to normalize *LPL* expression according to Pede et al. [14]. *LPL* mRNA expression level was measured in 192 patients.

To determine expression levels of *microRNA-410* (*miRNA-410*), *miRNA-410* specific cDNA was synthesized using the Taqman reverse transcription kit and specific stem-loop RT primers (Applied Biosystems, Life Technologies). PCR reactions were performed using an assay-on-demand hydrolysis probe based Taqman miRNA expression assay (Applied Biosystems, Life Technologies). For normalization, the two most stable small RNA controls (RNU 48, RNU 24) were used [18]. All qPCR reactions were performed in duplicate on a LightCycler 480 (Roche Diagnostics).

### LPL ELISA

To determine LPL protein levels in 44 samples, patient PBMCs were lysed using a 2× concentrated lysis buffer composed of 0.25% Triton X-100 (MP Biomedicals, Solon, Ohio, USA), 50 mM KCL, 100 mM TrisHCL pH 7.4, 40% glycerol [19]. The total protein concentration was determined using the Bio-Rad Bradford protein assay (Bio-Rad Laboratories, Nazareth, Belgium). 100 μl cellular lysate was used in a human LPL enzyme-linked immunosorbent assay (ELISA) (Cusabio, Wuhan, China) following manufacturer’s instructions.

### Fluorometric Lipase Activity Assay

Lipase activity of LPL was assessed by using the Confluolip fluorometric lipase activity assay (Progen, Heidelberg, Germany) according to the manufacturer’s instructions. Purified LPL from bovine milk (Sigma-Aldrich) served as a positive control.

### Statistical Analysis

All statistical analyses were performed with the GraphPad statistical software (GraphPad Software, La Jolla, CA, USA). All statistical tests were two-sided and an effect was considered statistically significant at *p* value < 0.05. ROC curve analysis was performed to determine the *LPL* mRNA expression cut-off value that best distinguished between mutated and unmutated cases. Median levels of different markers were compared between two groups using Mann-Whitney non parametric tests. Associations between different clinical markers were described with Pearson χ^2^ statistics (with the Yates continuity correction for 2 × 2 tables) or by calculating a Spearman's rank correlation coefficient. We used the Kaplan Meier method to analyze OS and TFS. The log-rank test was used to determine significant associations between individual markers and OS or TFS.

## Results

### Characterization of the Patient Cohort

The clinical and biological characteristics of the 248 CLL patients included in this study are detailed in Table 2. Median age at diagnosis is 62 years (range 34–86 years) and the male:female ratio is 2:1. The median TFS is 40 months (range 0–240 months) while the median OS is 78 months (range 1.5–240 months). 39 CLL-related deaths were observed during the observation period.

**Table 2:** Clinical and biological characteristics among *LPL* rs301, rs328 and rs13702 Genotypes.

	rs301 TT		rs301 TC/CC		
Characteristic	n	%	n	%	*p* ^*a*^
*No of patients*	146	59.1	101	40.9	
*HWE* ^*c*^		58.0		42.0	0.322
**Gender**					
Male	100	59.2	69	40.8	1.000
Female	46	59.0	32	41.0	
**Median age at diagnosis**	63		62		0.651^*b*^
**Age**					
<60 years	59	60.8	38	39.2	0.786
≥60 years	85	58.2	61	41.8	
**Binet stage**					
A	106	57.9	77	42.1	0.404
B/C	31	66.0	16	34.0	
***IGHV***					
Mutated	74	58.7	52	41.3	0.972
Unmutated	48	60.0	32	40.0	
**CD38**					
<7%	68	54.8	56	45.2	0.224
≥7%	65	63.7	37	36.3	
**ZAP70**					
<20%	75	60.5	49	39.5	1.000
≥20%	54	61.4	34	38.6	
***LPL***					
negative	62	63.3	36	36.7	0.821
positive	57	60.6	37	39.4	
**LDT**					
≥ 1 year	71	61.2	45	38.8	0.662
< 1 year	55	57.3	41	42.7	
**Cytogenetics**					
normal	36	61.0	23	39.0	0.590
chromosomal aberrations	87	55.8	69	44.2	
**Del13q**					
absent	68	63.6	39	36.4	0.232
present	51	54.3	43	45.7	
**Del11q**					
absent	109	58.3	78	41.7	0.606
present	11	50.0	11	50.0	
**Del17p**					
absent	106	55.5	85	44.5	0.563
present	13	65.0	7	35.0	
	**rs328 CC**		**rs328 CG/GG**		
**Characteristic**	**n**	**%**	**n**	**%**	***P*** ^***a***^
No of patients	195	78.6	53	21.4	
HWE^*c*^		77.9		22.2	0.123
**Gender**					
Male	134	78.8	36	21.2	1.000
Female	61	78.2	17	21.8	
**Median age at diagnosis**	62		62		0.878^*b*^
**Age**					
<60 years	79	80.6	19	19.4	0.752
≥60 years	114	78.1	32	21.9	
**Binet stage**					
A	144	78.3	40	21.7	0.610
B/C	39	83.0	8	17.0	
***IGHV***					
Mutated	104	81.9	23	18.1	0.421
Unmutated	61	76.3	19	23.8	
**CD38**					
<7%	99	79.8	25	20.2	0.954
≥7%	81	78.6	22	21.4	
**ZAP70**					
<20%	99	79.2	26	20.8	0.867
≥20%	68	77.3	20	22.7	
***LPL***					
negative	83	84.7	15	15.3	0.483
positive	75	79.8	19	20.2	
**LDT**					
≥ 1 year	92	79.3	24	20.7	1.000
< 1 year	77	79.4	20	20.6	
**Cytogenetics**					
Normal	46	78.0	13	22.0	1.000
Chromosomal aberrations	123	78.3	34	21.7	
**Del13q**					
absent	85	78.7	23	21.3	1.000
present	74	78.7	20	21.3	
**Del11q**					
absent	150	79.8	38	20.2	0.144
present	14	63.6	8	36.4	
**Del17p**					
Absent	149	77.6	43	22.4	0.316
Present	18	90.0	2	10.0	
	**rs13702 TT**		**rs13702 TC/CC**		
**Characteristic**	**n**	**%**	**n**	**%**	***p*** ^***a***^
No of patients	123	50.0	123	50.0	
HWE^*c*^		49.1		50.9	0.413
**Gender**					
Male	87	51.5	82	48.5	0.582
Female	36	46.8	41	53.2	
**Median age at diagnosis**	63		62		0.501^*b*^
**Age**					
<60 years	48	49.5	49	50.5	1.000
≥60 years	73	50.3	72	49.7	
**Binet stage**					
A	87	47.8	95	52.2	0.202
B/C	28	59.6	19	40.4	
***IGHV***					
Mutated	61	48.8	64	51.2	0.369
Unmutated	45	56.3	35	43.8	
**CD38**					
<7%	58	47.5	64	52.5	0.551
≥7%	54	52.4	49	47.6	
**ZAP70**					
<20%	62	50.0	62	50.0	0.663
≥20%	47	54.0	40	46.0	
***LPL***					
negative	49	50.5	48	49.5	0.877
positive	49	52.7	44	47.3	
**LDT**					
≥ 1 year	61	53.5	53	46.5	0.298
< 1 year	44	45.4	53	54.6	
**Cytogenetics**					
Normal	29	50.0	29	50.0	1.000
Chromosomal aberrations	77	49.4	79	50.6	
**Del13q**					
absent	55	51.4	52	48.6	0.968
present	49	52.7	44	47.3	
**Del11q**					
absent	96	51.6	90	48.4	0.126
present	7	31.8	15	68.2	
**Del17p**					
absent	93	48.9	97	51.1	0.920
present	9	45.0	11	55.0	

^a^ Cross-tabulations of prognostic markers versus *LPL* SNP genotypes. *p* values of Pearson χ² statistics (with the Yates continuity correction for 2x2 tables)

^b^ *p* value of Mann-Whitney non parametric test comparing median age at diagnosis between *LPL* SNP genotypes

^c^ HWE, Hardy-Weinberg equilibrium

Three SNPs in the *LPL* gene listed in Table 1 were examined. *LPL* rs301 is located in the non-coding region of intron 6 and results in a T<C nucleotide change. *LPL* rs328, involving a C<G nucleotide change in exon 9, inserts a nonsense mutation leading to a change in amino acid 447 from a serine to an early stop codon. The third *LPL* SNP, rs13702 is located in the 3’UTR of the *LPL* gene. This nucleotide change disrupts a miRNA recognition element (MRE) seed site (MRESS) for the human *miRNA-410* [20].

Of all SNPs, MAFs were calculated and genotype frequencies were determined to be in accordance with Hardy-Weinberg equilibrium (Table 1). The observed MAFs were comparable to expected frequencies in the Caucasian population, information extracted from the HapMap database (available: http://www.ncbi.nlm.nih.gov/). There was no difference in median age at diagnosis nor in male:female ratio for patients with wild-type or SNP alleles for all three examined *LPL* SNPs.

### *LPL* SNPs are Prognostic Markers in CLL

Chi-square tests showed significant associations between *IGHV* mutation status and its surrogate markers, CD38 status (*p* = 0.002), ZAP70 status (*p*<0.0001) and *LPL* status (*p*<0.0001) (see S1 Fig.). In this patient population no correlation was found between the examined *LPL* SNP genotypes and age at diagnosis, gender, Binet stage or *IGHV* mutation status. Furthermore no correlation was observed between CD38 or ZAP70 protein expression nor between the presence or absence of chromosomal aberrations and presence of either three SNP variants (Table 2).

**Fig 1:**
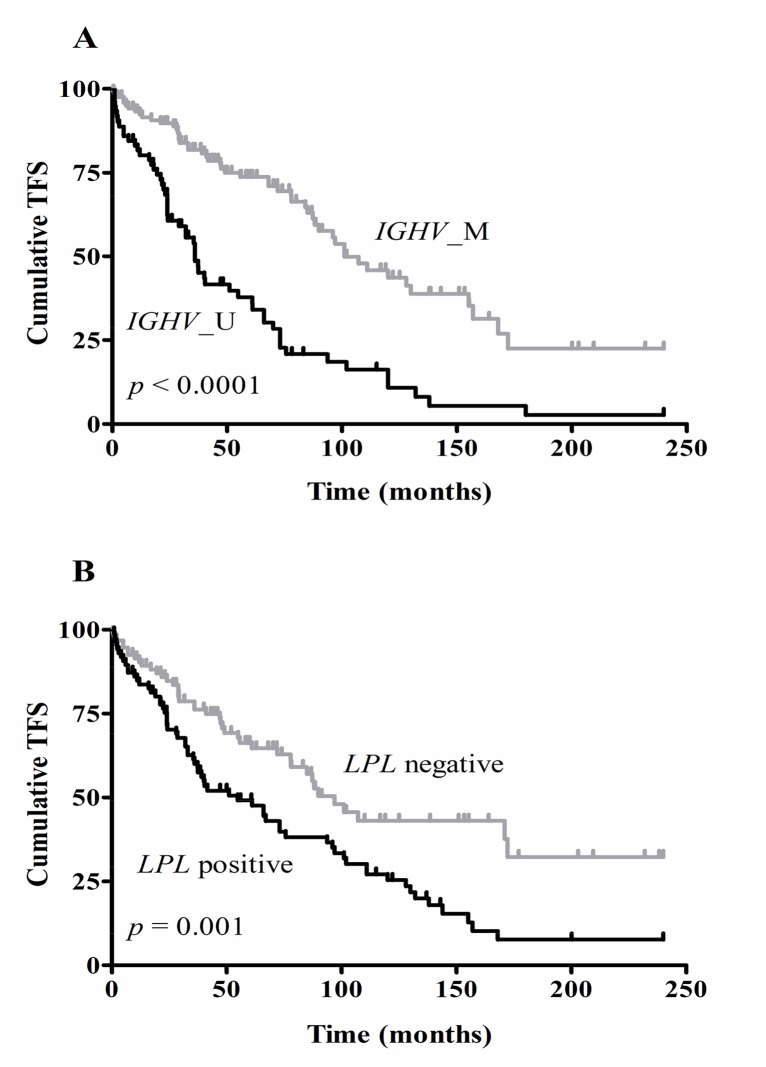
Kaplan Meier survival curves for TFS with regard to *IGHV* mutation status (A) and LPL mRNA expression (B). *IGHV* gene mutation status was based on a 98% cut-off value (n = 207; M, mutated; U, unmutated). Differentiation between *LPL* positive and negative cases was based on the optimal cut-off value determined by ROC curve analysis (n = 192). Log-rank tests showed significantly different TFS curves for *IGHV* mutation status (*p*<0.0001) and *LPL* mRNA expression (*p* = 0.001).

Log-rank tests showed a significant association between OS and Binet stage (*p* = 0.002), *IGHV* mutation status (*p*<0.0001), CD38 (*p* = 0.04) and ZAP70 protein expression (*p* = 0.006). The presence of the adverse chromosomal aberration del17p was also significantly associated with OS (*p* = 0.008). All prognostic markers were significantly associated with TFS, except presence or absence of del13q and del11q (see Table 3). Kaplan-Meier survival curves for TFS with regard to *IGHV* mutation status (1A) and *LPL* expression (1B) are shown in Fig. 1.

**Table 3:** Survival data for different biological and clinical characteristics and *LPL* SNPs.

Characteristic	Median OS^*b*^	p^*a*^	Median TFS^*b*^	p^*a*^
**Binet stage**				
A	UD^*c*^	0.002	87.2	< 0.0001
B/C	137.2		29.0	
***IGHV***				
Mutated	UD	< 0.0001	101.0	< 0.0001
Unmutated	165.0		36.0	
**CD38**				
<7%	UD	0.042	97.0	<0.0001
≥7%	UD		40.4	
**ZAP70**				
<20%	UD	0.006	97.0	< 0.0001
≥20%	170.0		36.0	
***LPL***				
Negative	UD	0.282	89.9	0.001
Positive	UD		41.6	
**LDT**				
≥ 1 year	UD	0.167	88.1	0.001
< 1 year	UD		41.6	
**Cytogenetics**				
Normal	UD	0.124	96.0	0.016
Chromosomal aberrations	UD		61.0	
**Del13q**				
absent	UD	0.168	67.0	0.748
present	UD		73.0	
**Del11q**				
absent	UD	0.854	73.0	0.244
present	UD		36.0	
**Del17p**				
absent	UD	0.008	75.7	0.016
present	133.0		26.7	
***LPL*rs301**				
TT	UD	0.026	72.0	0.953
TC/CC	UD		93.7	
***LPL*rs328**				
CC	UD	0.483	75.7	0.477
CG/GG	UD		67.0	
***LPL*rs13702**				
TT	UD	0.008	66.0	0.678
TC/CC	UD		87.2	

^a^ *p* values of log-rank tests

^b^ Survival curves for overall survival (OS) and treatment free survival (TFS) were estimated by the Kaplan-Meier method

^c^ UD, undetermined, indicating that the median value was not reached

**Fig 2:**
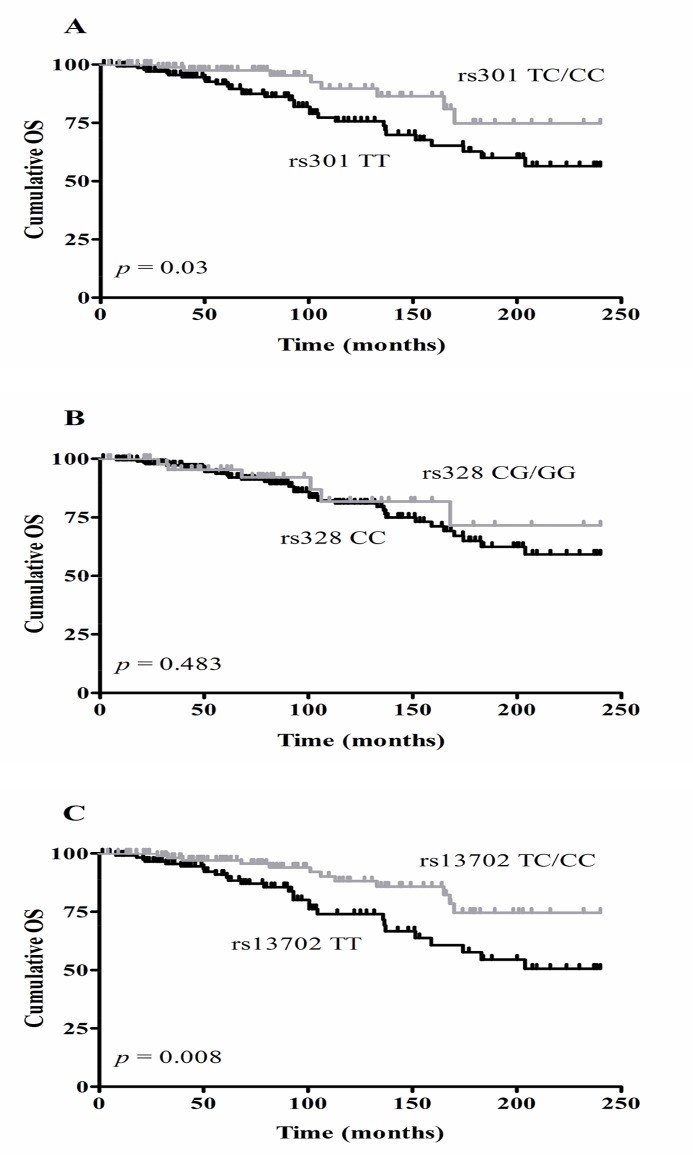
Kaplan-Meier curves for OS according to *LPL* SNP rs301 (A), rs328 (B) and rs13702 (C) genotypes. A significant effect on OS was indicated by Log-rank tests for rs301 (n = 247; *p* = 0.03) and for rs13702 (n = 246; *p* = 0.008), but not for rs328 (n = 248; p = 0.483).

Although none of the examined SNPs correlated significantly with well-known prognostic markers in CLL, two of them, rs301 and rs13702, were significantly associated with OS (Log-rank test; *p* = 0.03 and *p* = 0.008 respectively). Indeed, patients being either heterozygous or homozygous for these SNPs showed a better outcome in terms of OS compared to patients having the wild-type genotype. This was not the case for rs328 (*p* = 0.483) and notably none of the SNPs affected TFS significantly. Fig. 2 shows the Kaplan Meier curves for OS.

### *LPL* SNPs and LPL expression

GWAS have reported several genetic variants in the *LPL* gene affecting lipid profile levels, among them the *LPL* SNPs analyzed in present study. For *LPL* rs301 a direct effect on LPL expression or activity was not reported yet. Rs328 and rs13702 however are extensively studied gain-of-function variants, known to increase LPL activity.

**Fig 3:**
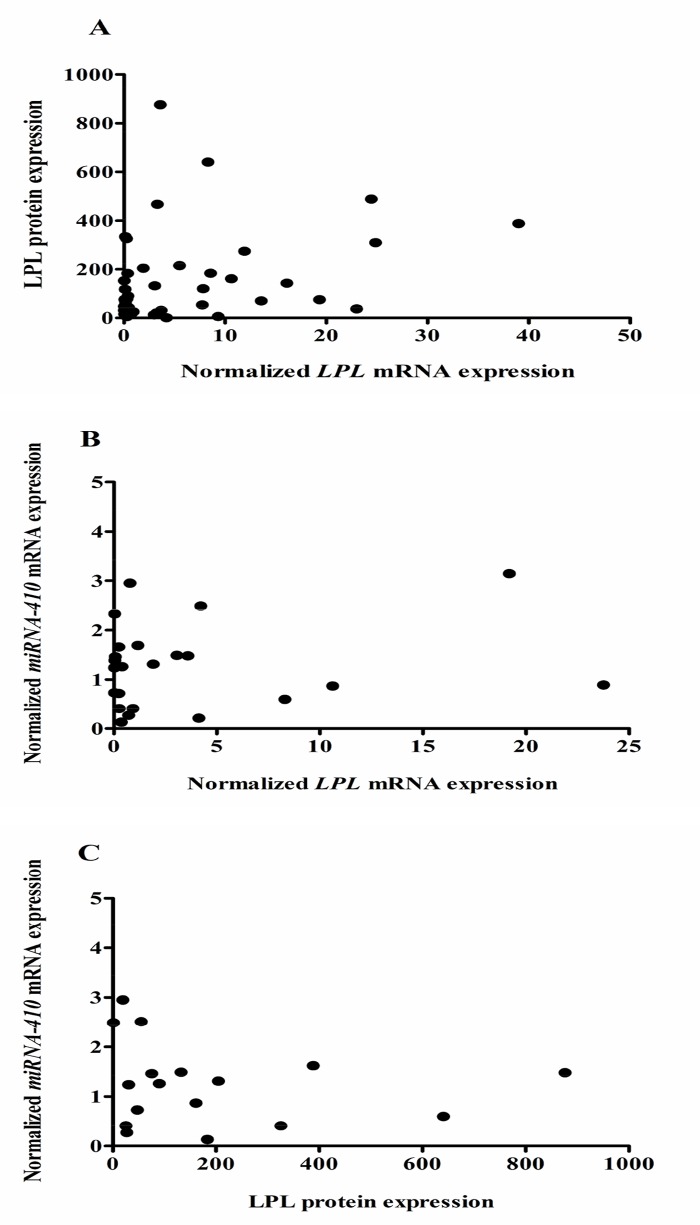
Correlation between LPL protein expression and *LPL* mRNA expression (A) and between *miRNA-410* expression and *LPL* mRNA (B) or LPL protein (C) expression. *MiRNA-410* mRNA (n = 25) and *LPL* mRNA (n = 92) expression levels were determined by qPCR analysis, LPL protein levels were determined by ELISA (n = 44). No correlation was found between LPL protein and mRNA levels (Spearman’s rank correlation coefficient = 0.29) (A). No significant correlation between *miRNA-410* expression and LPL mRNA (B) or protein levels (C) could be observed.

Since two of these SNPs were significantly associated with outcome of CLL patients in this cohort, we wanted to evaluate whether these SNPs affected LPL expression levels and lipase activity. For all three variants examined we couldn’t find a significant difference in *LPL* mRNA expression levels between patients carrying the wild-type allele or the SNP allele (rs301; *p* = 0.94, rs328; *p* = 0.64, rs13702; *p* = 0.67). LPL protein levels did not correlate with *LPL* mRNA expression levels (Spearman’s rank correlation coefficient = 0.29, Fig. 3A). Comparable to what was found on mRNA level, we couldn’t find a significant association between LPL protein levels and presence of any of the *LPL* SNPS (rs301; *p* = 0.44, rs328; *p* = 0.56, rs13702; *p* = 0.58). Finally, we also investigated whether there is a difference in lipase activity between samples of patients carrying one of the SNP alleles or not. As published before by others [21], we could not, or only at a very low level, detect lipase activity in CLL cell lysates and no further analyses were performed.

The *LPL* SNP rs13702 was shown to modulate lipid levels through disruption of a binding site in the 3’UTR of the *LPL* gene for the human *miRNA-410* resulting in a gain-of-function variant [20]. To see whether this *miRNA-410* affects LPL expression in CLL cells as well, we determined its expression in 25 CLL patient samples with varying LPL mRNA and protein levels. Overall the expression levels of this miRNA in the selected CLL samples were very low and no significant association with LPL mRNA or protein levels could be observed (Fig. 3B and 3C), although the latter showed a trend of inverse correlation.

## Discussion

In the present study we examined three SNPs in the *LPL* gene, rs301, rs328 and rs13702, in a cohort of 248 CLL patients and found that rs301 and rs13702 affected OS significantly, whereas no association with OS could be observed for rs328.

Based on these findings, we also investigated whether the *LPL* SNP genotypes correlated with other well known prognostic markers in this CLL patient population. Notably, no association with the *IGHV* mutation status, the gold standard in CLL prognostication, could be observed. This indicates that the SNPs rs301 and rs13702, showing a significant correlation with OS, are no surrogate markers for the *IGHV* mutation status, but could function as independent prognostic markers in CLL. In line with these results none of the SNPs showed a correlation with other prognostic markers such as ZAP70, CD38 or FISH. This independency was highlighted by Malek as one of the reasons positively affecting the applicability of a CLL biomarker [22]. However, in a multivariate analysis including age at diagnosis, *IGHV*, ZAP70, *LPL*, CD38, del11q, del17p and the three *LPL* SNPs, only age, ZAP70 and del17p were significant and independent predictors of OS (age; *p* = 0.003, ZAP70; *p* = 0.009, del17p; *p* = 0.02). We could not show that the *LPL* SNPs have additional prognostic value, which is probably due to a lack of power in our study.

Other characteristics of a perfect CLL biomarker that are fulfilled for the SNPs we describe, include the use of a widely available, reliable and valid measuring technique, the fact that genotyping can be performed in peripheral blood samples, the stability of the SNP status over time and the fact that these SNP markers don’t require the use of arbitrary cut-off values for marker positivity, unlike most currently available biomarkers. Moreover, in case no RNA or protein is available, this DNA based marker can still provide prognostic information. This is in contrast with the labor-intensive procedure required for *IGHV* mutation status analysis or the ZAP70 analysis which is extremely difficult to standardize.

A major shortcoming of these SNP prognostic markers is the fact that these *LPL* SNPs, as far as we know now, do not represent a biological mechanism directly involved in CLL cell biology. For LPL mRNA or protein however such a relationship with an intrinsic feature of the leukemic cells was not identified either.

The *LPL* SNPs rs301 and 13702 affected OS significantly, but did not correlate with TFS in this CLL patient cohort, indicating that disease progression is not affected. We speculate that CLL patients carrying these SNPs are in an overall better shape and are less vulnerable to infections or respond better to therapy, explaining the observed association with better disease outcome. These genotypes might not affect disease outcome or therapy responsiveness due to an intrinsic effect on CLL cell biology, but could affect CLL comorbidity. Since CLL is mainly a disease of the elderly, patients are often compromised by co-existent pathological conditions or deterioration of their overall health. In a study investigating the effect of CLL on the quality of life, 71.5% of 1482 patients that were included had at least one co-morbid health problem, with hypertension and increased cholesterol being the most frequent comorbidities [23]. Both conditions were demonstrated to be affected by the presence of the *LPL* SNPs under study [12,13]. Indeed these SNPs were found to be among the list of *LPL* variants positively affecting ‘healthy’ HDL cholesterol (HDL-C) and triglyceride (TG) levels, being important risk factors for these and other pathological conditions [12,13]. The mechanism by which rs301 affects HDL-C and TG levels is unknown yet, for rs328 and rs13702 however possible explanations for the observed phenotypes were reported. The *LPL* SNP rs13702 was shown to modulate lipid levels through disruption of a MRESS in the 3’UTR of the *LPL* gene for the human *miRNA-410*. Richardson et al. found that while in the majority of the people binding of *miRNA-410* to the *LPL* mRNA reduced synthesis of LPL, carriers of the genetic variant rs13702 showed no miRNA activity, presumably higher LPL levels and as a consequence lower TG levels and higher HDL-C levels [20]. Also for rs328 a mechanism has been described by which this variant contributes to favorable lipid profiles and reduces the risk for common disease. This nucleotide polymorphism in exon 9 of the *LPL* gene, introduces a premature stop codon resulting in a truncated protein with a higher activity giving a comparable phenotype (lower TG, higher HDL-C) as observed for rs13702 carriers [24].

Despite the observed effect of rs13702 and rs328 on lipase activity reported by others, we couldn’t find an association between presence of any of these SNPs and LPL mRNA or protein levels, suggesting that LPL expression is controlled by other not yet defined mechanisms in CLL cells. We also tried to compare the lipase activity between CLL samples with different genotypes for the SNPs under study, but overall lipase activity was very low to undetectable, which is in accordance with previous findings [21]. However, the biological role of LPL in CLL remains to be elucidated, indicating that a not yet defined mechanism could be responsible for the observed results as well.

The *LPL* SNPs that we evaluated in this study display important features that other currently available markers often lack, making them reliable prognostic markers that could help to improve the management of CLL. In this respect, it would be interesting to evaluate the clinical utility, stability and robustness of these genetic prognostic indicators, alone or in combination with each other and other clinical markers in large patient cohorts [25].

## Supporting Information

S1 Fig Correlation between CD38 (A), ZAP70 (B) or *LPL* (C) expression and *IGHV* status. CD38 (n = 218) and ZAP70 (n = 167) protein expression were determined by flow cytometry, *LPL* mRNA expression was determined by qPCR analysis (n = 92). *IGHV* gene mutation status was based on a 98% cut-off value (n = 207). Mann-Whitney non parametric tests showed statistically significant differences between the median CD38 (*p* = 0.002), ZAP70 (*p*<0.0001) and *LPL* (*p*<0.0001) expression levels in *IGHV* mutated (M) and unmutated (U) CLL cases.
(TIF)
