# Correction: Lipoprotein Lipase SNPs rs13702 and rs301 Correlate with Clinical Outcome in Chronic Lymphocytic Leukemia Patients

**DOI:** 10.1371/journal.pone.0131029

**Published:** 2015-06-16

**Authors:** The PLOS ONE Staff

In Table 2, in the PDF version of the article, the first row is incorrectly replicated at the beginning of each new page. Please see the corrected Table 2 here.

**Table 2:** Clinical and biological characteristics among *LPL* rs301, rs328 and rs13702 Genotypes.

** **	**rs301 TT**		**rs301 TC/CC**		** **
**Characteristic**	**n**	**%**	**n**	**%**	***p*** ^***a***^
No of patients	146	59.1	101	40.9	
HWE^*c*^		58.0		42.0	0.322
**Gender**					
Male	100	59.2	69	40.8	1.000
Female	46	59.0	32	41.0	
**Median age at diagnosis**	63		62		0.651^*b*^
**Age**					
<60 years	59	60.8	38	39.2	0.786
≥60 years	85	58.2	61	41.8	
**Binet stage**					
A	106	57.9	77	42.1	0.404
B/C	31	66.0	16	34.0	
***IGHV***					
Mutated	74	58.7	52	41.3	0.972
Unmutated	48	60.0	32	40.0	
**CD38**					
<7%	68	54.8	56	45.2	0.224
≥7%	65	63.7	37	36.3	
**ZAP70**					
<20%	75	60.5	49	39.5	1.000
≥20%	54	61.4	34	38.6	
***LPL***					
negative	62	63.3	36	36.7	0.821
positive	57	60.6	37	39.4	
**LDT**					
≥ 1 year	71	61.2	45	38.8	0.662
< 1 year	55	57.3	41	42.7	
**Cytogenetics**					
normal	36	61.0	23	39.0	0.590
chromosomal aberrations	87	55.8	69	44.2	
**Del13q**					
absent	68	63.6	39	36.4	0.232
present	51	54.3	43	45.7	
**Del11q**					
absent	109	58.3	78	41.7	0.606
present	11	50.0	11	50.0	
**Del17p**					
absent	106	55.5	85	44.5	0.563
present	13	65.0	7	35.0	
** **	**rs328 CC**		**rs328 CG/GG**		** **
**Characteristic**	**n**	**%**	**n**	**%**	***P*** ^***a***^
No of patients	195	78.6	53	21.4	
HWE^*c*^		77.9		22.2	0.123
**Gender**					
Male	134	78.8	36	21.2	1.000
Female	61	78.2	17	21.8	
**Median age at diagnosis**	62		62		0.878^*b*^
**Age**					
<60 years	79	80.6	19	19.4	0.752
≥60 years	114	78.1	32	21.9	
**Binet stage**					
A	144	78.3	40	21.7	0.610
B/C	39	83.0	8	17.0	
***IGHV***					
Mutated	104	81.9	23	18.1	0.421
Unmutated	61	76.3	19	23.8	
**CD38**					
<7%	99	79.8	25	20.2	0.954
≥7%	81	78.6	22	21.4	
**ZAP70**					
<20%	99	79.2	26	20.8	0.867
≥20%	68	77.3	20	22.7	
***LPL***					
negative	83	84.7	15	15.3	0.483
positive	75	79.8	19	20.2	
**LDT**					
≥ 1 year	92	79.3	24	20.7	1.000
< 1 year	77	79.4	20	20.6	
**Cytogenetics**					
Normal	46	78.0	13	22.0	1.000
Chromosomal aberrations	123	78.3	34	21.7	
**Del13q**					
absent	85	78.7	23	21.3	1.000
present	74	78.7	20	21.3	
**Del11q**					
absent	150	79.8	38	20.2	0.144
present	14	63.6	8	36.4	
**Del17p**					
absent	149	77.6	43	22.4	0.316
present	18	90.0	2	10.0	
** **	**rs13702 TT**		**rs13702 TC/CC**		** **
**Characteristic**	**n**	**%**	**n**	**%**	***p*** ^***a***^
No of patients	123	50.0	123	50.0	
HWE^*c*^		49.1		50.9	0.413
**Gender**					
Male	87	51.5	82	48.5	0.582
Female	36	46.8	41	53.2	
**Median age at diagnosis**	63		62		0.501^*b*^
**Age**					
<60 years	48	49.5	49	50.5	1.000
≥60 years	73	50.3	72	49.7	
**Binet stage**					
A	87	47.8	95	52.2	0.202
B/C	28	59.6	19	40.4	
***IGHV***					
Mutated	61	48.8	64	51.2	0.369
Unmutated	45	56.3	35	43.8	
**CD38**					
<7%	58	47.5	64	52.5	0.551
≥7%	54	52.4	49	47.6	
**ZAP70**					
<20%	62	50.0	62	50.0	0.663
≥20%	47	54.0	40	46.0	
***LPL***					
negative	49	50.5	48	49.5	0.877
positive	49	52.7	44	47.3	
**LDT**					
≥ 1 year	61	53.5	53	46.5	0.298
< 1 year	44	45.4	53	54.6	
**Cytogenetics**					
Normal	29	50.0	29	50.0	1.000
Chromosomal aberrations	77	49.4	79	50.6	
**Del13q**					
absent	55	51.4	52	48.6	0.968
present	49	52.7	44	47.3	
**Del11q**					
absent	96	51.6	90	48.4	0.126
present	7	31.8	15	68.2	
**Del17p**					
absent	93	48.9	97	51.1	0.920
present	9	45.0	11	55.0	

^a^ Cross-tabulations of prognostic markers versus *LPL* SNP genotypes. *p* values of Pearson χ^2^ statistics (with the Yates continuity correction for 2x2 tables)

^b^ *p* value of Mann-Whitney non parametric test comparing median age at diagnosis between *LPL* SNP genotypes

^c^ HWE, Hardy-Weinberg equilibrium

Table 3 has been corrected for improved readability. Please see the corrected Table 3 here.

**Table 3:** Survival data for different biological and clinical characteristics and *LPL* SNPs.

Characteristic	Median OS^*b*^	*p* ^*a*^	Median TFS^*b*^	*p* ^*a*^
**Binet stage**				
A	UD^*c*^	0.002	87.2	< 0.0001
B/C	137.2		29.0	
***IGHV***				
Mutated	UD	< 0.0001	101.0	< 0.0001
Unmutated	165.0		36.0	
**CD38**				
<7%	UD	0.042	97.0	<0.0001
≥7%	UD		40.4	
**ZAP70**				
<20%	UD	0.006	97.0	< 0.0001
≥20%	170.0		36.0	
***LPL***				
Negative	UD	0.282	89.9	0.001
Positive	UD		41.6	
**LDT**				
≥ 1 year	UD	0.167	88.1	0.001
< 1 year	UD		41.6	
**Cytogenetics**				
Normal	UD	0.124	96.0	0.016
Chromosomal aberrations	UD		61.0	
**Del13q**				
absent	UD	0.168	67.0	0.748
present	UD		73.0	
**Del11q**				
absent	UD	0.854	73.0	0.244
present	UD		36.0	
**Del17p**				
absent	UD	0.008	75.7	0.016
present	133.0		26.7	
***LPL*rs301**				
TT	UD	0.026	72.0	0.953
TC/CC	UD		93.7	
***LPL*rs328**				
CC	UD	0.483	75.7	0.477
CG/GG	UD		67.0	
***LPL*rs13702**				
TT	UD	0.008	66.0	0.678
TC/CC	UD		87.2	

^a^ *p* values of log-rank tests

^b^ Survival curves for overall survival (OS) and treatment free survival (TFS) were estimated by the Kaplan-Meier method

^c^ UD, undetermined, indicating that the median value was not reached

The publisher apologizes for the errors.
