# Supplementary figures and images for: Lipoprotein Lipase SNPs rs13702 and rs301 Correlate with Clinical Outcome in Chronic Lymphocytic Leukemia Patients
Source: PLoS One. 2015 Mar 26;10(3):e0121526. doi: 10.1371/journal.pone.0121526 (PMC4374908; doi:10.1371/journal.pone.0121526)

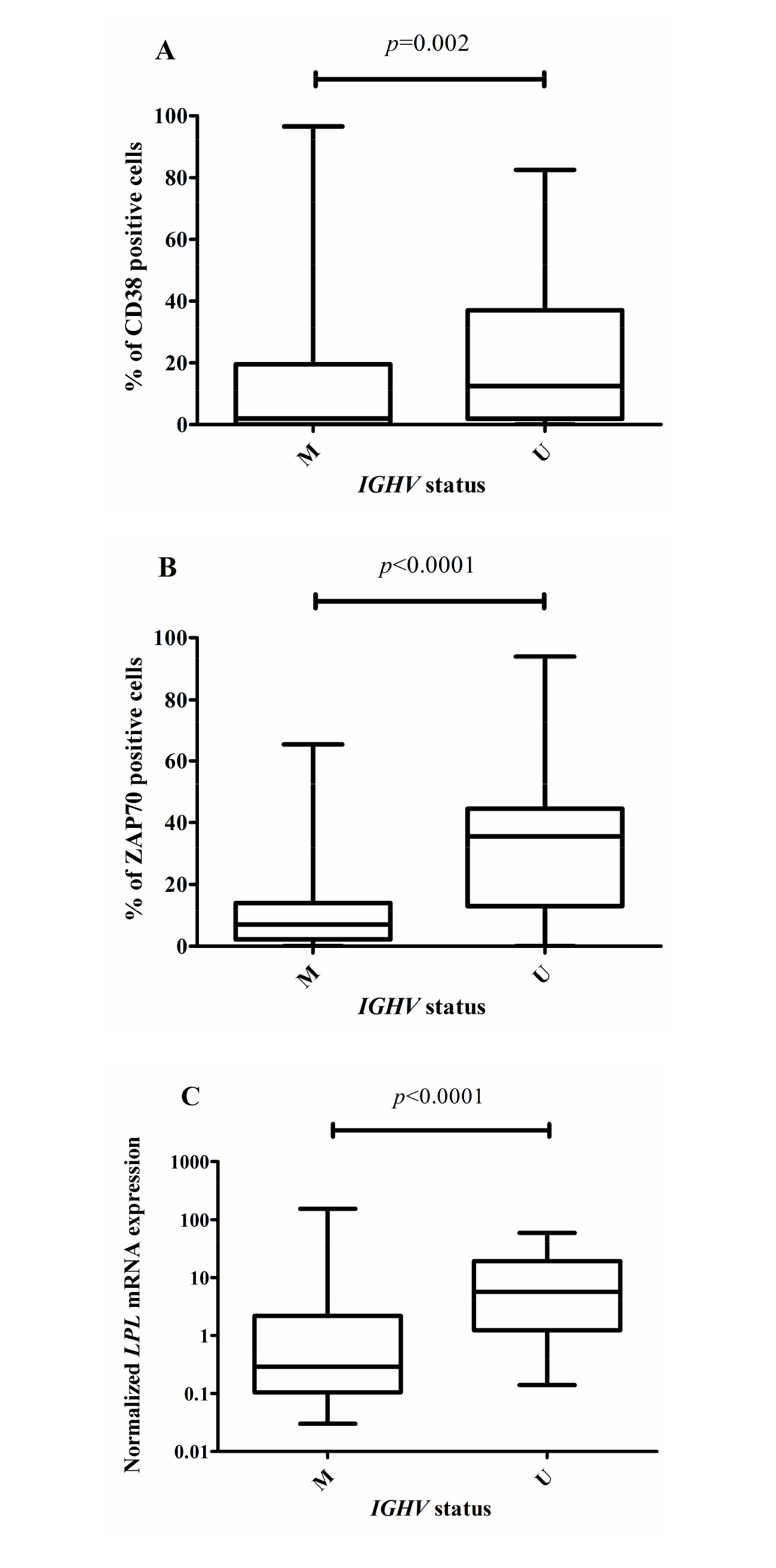

Supplement: S1 Fig — CD38 (n = 218) and ZAP70 (n = 167) protein expression were determined by flow cytometry, LPL mRNA expression was determined by qPCR analysis (n = 92). IGHV gene mutation status was based on a 98% cut-off value (n = 207). Mann-Whitney non parametric tests showed statistically significant differences between the median CD38 (p = 0.002), ZAP70 (p<0.0001) and LPL (p<0.0001) expression levels in IGHV mutated (M) and unmutated (U) CLL cases. (TIF) [file pone.0121526.s001.tif]
